# Frequency of NFKBIA deletions is low in glioblastomas and skewed in glioblastoma neurospheres

**DOI:** 10.1186/1476-4598-12-160

**Published:** 2013-12-11

**Authors:** Monica Patanè, Paola Porrati, Elisa Bottega, Sara Morosini, Gabriele Cantini, Vita Girgenti, Ambra Rizzo, Marica Eoli, Bianca Pollo, Francesca L Sciacca, Serena Pellegatta, Gaetano Finocchiaro

**Affiliations:** 1 Molecular Neuro-Oncology Unit, IRCCS Foundation “C.Besta” Neurological Institute, via Celoria 11, 20133 Milan, Italy; 2 Department of Experimental Oncology, IFOM-IEO Campus, via Adamello 16, 20139 Milan, Italy; 3 Unit of Clinical Pathology and Medical Genetics, IRCCS Foundation “C.Besta” Neurological Institute, via Celoria 11, 20133 Milan, Italy; 4 Neuropathology Unit, IRCCS Foundation “C.Besta” Neurological Institute, via Celoria 11, 20133 Milan, Italy

## Abstract

The NF-kB family of transcription factors is up-regulated in inflammation and different cancers. Recent data described heterozygous deletions of the NF-kB Inhibitor alpha gene (*NFKBIA*) in about 20% of glioblastomas (GBM): deletions were mutually exclusive with epidermal growth factor receptor (*EGFR*) amplification, a frequent event in GBM. We assessed the status of *NFKBIA* and *EGFR* in 69 primary GBMs and in corresponding neurospheres (NS). *NFKBIA* deletion was investigated by the copy number variation assay (CNV); *EGFR* amplification by CNV ratio with *HGF*; expression of *EGFR* and *EGFRvIII* by quantitative PCR or ReverseTranscriptase PCR. Heterozygous deletions of *NFKBIA* were present in 3 of 69 primary GBMs and, surprisingly, in 30 of 69 NS. *EGFR* amplification was detected in 36 GBMs: in corresponding NS, amplification was lost in 13 cases and reduced in 23 (10 vs 47 folds in NS vs primary tumors; p < 0.001). The CNV assay was validated investigating *HPRT1* on chromosome X in females and males. Results of array-CGH performed on 3 primary GBMs and 1 NS line were compatible with the CNV assay. NS cells with *NFKBIA* deletion had increased nuclear activity of p65 (RelA) and increased expression of the NF-kB target IL-6. In absence of EGF in the medium, *EGFR* amplification was more conserved and *NFKBIA* deletion less frequent point to a low frequency of *NFKBIA* deletions in GBM and suggest that EGF in the culture medium of NS may affect frequency not only of *EGFR* amplifications but also of *NFKBIA* deletions.

## Introduction

Glioblastoma multiforme (GBM) is the highest grade glioma, according to World Health Organization classification, and has an annual incidence of 5 cases per 100,000 people [1,2]. In recent years, a huge effort was made to achieve a more thorough characterization of genetic and molecular signatures of GBM, facilitating the identification of new molecular targets and leading to a classification in four molecular subtypes: classical, mesenchymal, proneural and neural [3,4]. The classical subtype is mostly characterized by loss of chromosome 10 and amplification of the epidermal growth factor receptor gene (*EGFR*).

In the past years several studies have pointed to the importance of EGFR and NF-kB pathways in formation, growth and relapse of many tumor types, including GBM, and recent evidence suggests a cross-talk between these pathways [5-8].

NF-kB is a heterodimeric transcription factor formed by a family of Rel proteins, sharing a common N-terminal DNA binding region (RelA/p65, RelB and RelC), and by proteins that contain an ankyrin domain (p50, p100). NF-kB complexes are maintained inactive in the cytoplasm through interaction with their inhibitor IkBα, encoded by the *NFKBIA* gene located on chromosome 14q13.2. Most stimuli activate this pathway through phosphorylation of the IKK complex, which in turn phosphorylates IkBα, leading the inhibitor to degradation and allowing nuclear translocation of NF-kB. In the nucleus, NF-kB regulates the transcription of several genes involved in proliferation, survival, tissue invasion, inhibition of apoptosis and angiogenesis, including several chemokines and cytokines [9]. Two pathways of NF-kB activation have been described: canonical and non-canonical, involving different types of kinases (STAT3, PI3K/Akt, MAPK) and distinct heterodimers (p65/p50; p100/RelB) [10].

EGFR is mostly involved in proliferation and is expressed at high levels in many types of cancers, including glioblastoma [11]. EGFR is a tyrosine-kinase receptor, which signals through two main pathways: Ras/MAPK kinases and PI3K/Akt/mTOR kinases. Its gene, located on chromosome 7p11.2, is amplified in ~40% GBMs, forming typical double-minutes, auto-replicative chromosomes: this amplification is distinguished from polisomy of chromosome 7, a frequent event in GBM. *EGFR* gene can be also mutated: in particular, the deletion of exons 2-7 generates a constitutively activated form, called EGFRvIII, which lacks of the extracellular domain and is not able to bind the EGF ligand and to internalize, leading to low-level continuous signalling. This mutant is present in ~ 50% of GBM with *EGFR* amplification [12,13] and reciprocal interactions between EGFR and EGFRvIII have been reported recently [14]. Another type of *EGFR* amplification was observed in ~ 30% of GBM with extra copies of the gene inserted in different loci of chromosome 7; in this form of amplification the number of gene copies is small and the percentage of amplified cells is less than 15% [15].

Several studies have pointed to a relationship between EGFR and NFKB pathways mostly through activation of PI3K/Akt/mTOR signalling [7,16,17]. Relevant interactions between EGFR, the most important oncogene in GBMs, and NF-kB have been first proposed in breast cancer [16]. In GBMs association between SHP-2 and Grb2-associated binder 1 (Gab1) was identified as a critical step in the pathway linking EGFR to NF-kB activation [18]. One report, in particular, proposed a correlation between NF-kB and *EGFR* status at the genetic level, describing the heterozygous deletion of *NFKBIA* in 20% of primary GBMs in mutual exclusion with *EGFR* amplification [19]. However, in a previous studiy based on single nucleotide polymorphism DNA microarray analysis of GBM we did not find evidence of chromosomal imbalance on chromosome 14q13.2, where *NFKBIA* is mapping [20].

We decided to investigate the *NFKBIA* status in relationship to *EGFR* in primary GBM and in GBM stem-like cells, a highly tumorigenic subpopulation exploiting stem cell programs for glioma perpetuation [21]. These cells are traditionally grown as neurospheres (NS) in the presence of EGF and bFGF, mimicking the growth of normal neural stem cells [22,23]. Surprisingly, we found that *NFKBIA* deletion is rare in primary tumors, but becomes frequent in GBM NS, an event that seems to be affected by the presence of EGF in the culture medium.

## Material and methods

### Tumor specimens and cell cultures

The research work described in this paper is in compliance with the Helsinki Declaration. Patients considered in this study had signed an informed consent, defined and approved by the Quality Office of the Istituto Neurologico Besta (CI 25), agreeing on the use of part of their tumor specimens for research purposes. Human glioblastoma specimens, diagnosed as grade IV gliomas according to WHO criteria, were snap frozen or paraffin-embedded after surgery. Neurospheres (here equivalent to glioblastoma stem-like cells) were derived by mechanical dissociation and digestion of tumor specimens with collagenase type I (Invitrogen, Life technologies, Foster City CA, USA). Single cell suspensions were plated at clonal density (50 cells/μl) in standard medium containing: DMEM/F-12 (GIBCO, Life Technologies, Foster City CA, USA), 2 mM glutamine (Sigma Aldrich, St.Louis MO, USA), penicillin-streptomycin (1:100, EuroClone, Italy), B-27 supplement (1:50, GIBCO, Life technologies, Foster City CA, USA), human recombinant fibroblast growth factor 2 (bFGF 20 ng/ml; Tebu Bio, France) and epidermal growth factor (EGF, 20 ng/ml; Tebu Bio, France).

### Nucleic acid extraction

Total DNA was extracted using QIAamp DSP DNA Blood Mini Kit (Qiagen, Hilden, Germany) for peripheral blood and PureLink™ Pro 96 Genomic DNA Purification Kit (Invitrogen, Life technologies, Foster City CA, USA) for paraffin-embedded tumors according to protocols of the manufacturers. Total DNA and RNA from neurospheres were extracted using All Prep DNA/RNA mini kit (Qiagen, Hilden, Germany). RNA from snap frozen specimens was extracted by TRIZOL® (Invitrogen, Life technologies, Foster City CA, USA) using the manufacturer’s protocol. DNA and RNA concentration and purity were measured using Nanodrop® (Thermo Scientific, Pierce Biotechnology, Rockford IL, USA).

### Copy number variation assay

Copy number variation assay was performed on ViiA™ 7 Real-Time PCR System (Applied Biosystems, Life technologies, Foster City CA, USA) using Taqman® copy number assay chemistry (Applied Biosystems, Life technologies, Foster City CA, USA). Duplex real-time PCR was performed using 5 ng/μl of DNA. FAM-labeled assays were used for *EGFR* (Hs4942325_cn), *HGF* (Hs04982672_cn), *NFKBIA* (Hs01379535_cn), *HPRT1* (Hs05654996_cn) (Applied Biosystems, Life technologies, Foster City CA, USA) and VIC-labeled assay was used for reference gene (*TERT* 20X, Applied Biosystems, Life technologies, Foster City CA, USA). DNA extracted from peripheral blood was used as control. To discriminate between *EGFR* amplification and polysomy of chromosome 7, *EGFR* copy number was normalized against *HGF* copy number also mapping on chr7 and usually not amplified: threshold for amplification was *EGFR/HGF* copy number ratio ≥ 4.

### RT-PCR and real time PCR

500 ng RNA were reverse-transcribed using the High Capacity cDNA Reverse Transcription kit on 2720 Thermal Cycler (Applied Biosystems, Life technologies, Foster City CA, USA).

Real Time PCR was performed using Taqman® reagents on ViiA™ 7 Real-Time PCR System (Applied Biosystems, Life technologies, Foster City CA, USA): Taqman Universal Master Mix, *EGFR* assay (Hs_01076078_m1) and *GAPDH* assay as calibrator (Hs_99999905_m1) (Applied Biosystems, Life technologies, Foster City CA, USA). FirstChoice® Human Brain Total RNA was used as control (Ambion, Life technologies, Foster City CA, USA).

Amplification of full length *EGFR* and EGFRvIII was performed using Taq Gold® DNA Polymerase on 2720 Thermal Cycler (Invitrogen, Life technologies, Foster City CA, USA). PCR conditions were as follows: 95°C for 10 min; 42 × [95°C for 30 sec, 56.5°C for 30 sec, and 72°C for 1 min 20 sec sec]; and 72°C for 10 min. PCR products were analyzed into a 2.0% agarose gel. The following primers were used: EGFRvIII forward 5′-CTTCGGGGAGCAGCGATGCGA-3′ and reverse 5′-ACCAATACCTATTCCGTTACA-3′; wtEGFR forward 5′-CCAGTATTGATCGGGAGAGC-3′ and reverse 5′- CCAAGGACCACCTCACAGTT-3′ (PRIMM, Italy).

### Evaluation of NF-kB activity and IL-6 levels

Cytoplasmatic and nuclear proteins were extracted using the Nuclear extract kit (Active Motif, Carlsbad CA, USA) according to manufacturer’s protocol. Protein concentration was measured by MicroBCA Protein Assay kit at OD = 540 nm (Thermo Scientific, Pierce Biotechnology, Rockford IL, USA) and analyzed using a microplate absorbance reader Multiskan FC (Thermo Scientific, Pierce Biotechnology, Rockford IL, USA).

NF-kB activity was measured in protein extracts using the TransAM^TM^ NF-kB p65 protein assay (Active Motif, Carlsbad CA, USA), an ELISA-based method in which an oligonucleotide representing the NF-kB consensus binding site is immobilized on 96-well plates; p65 subunit is revealed by HRP-conjugated antibodies and analyzed at OD = 450 nm.

Interleukin-6 levels were measured from supernatants of neurospheres using Human IL-6 ELISA kit 96-well plate, based on standard sandwich enzyme-linked immune-adsorbent technology and revealed by Avidin-Biotin-Peroxidase Complex System (Wuhan Boster Biological Technology, Wuhan China), according to manufacturer’s protocol and analyzed at OD = 450 nm.

### Array-CGH

Array-CGH was performed using the CytoChip oligo-array (ISCA) 180K, containing 181,873 oligonucleotide probes (BlueGnome Ldt, Cambridge, UK) with a mean resolution of 16.30 Kb (25 Kb resolution on the backbone, 3.4 Kb resolution on genes). Data analysis was performed using InnoScan 710 Microarray Scanner (Innopsys Inc. Chicago IL, USA) and Bluefuse software (BlueGnome Ldt, Cambridge, UK). Oligo positions are referred to hg19.

One μg of sample and reference genomic DNAs were digested and then labelled with Cy3 (samples) and Cy5 (references) fluorophores, using random primers. The labelled DNAs were cleaned up using Amicon Ultracel-30 membrane filters (AU-30) (Millipore, Billerica MA, USA) followed by vacuum concentration if required. Labelled DNAs of samples were combined with sex-matched labeled references and then hybridized with oligonucleotide probes of array platform. Hybridization was performed using MaiTaiTM Hybridization System (SciGene, Sunnivale CA, USA). After 24 hours CytoChip oligo-array was washed and scanned. Amplifications or deletions are revealed by green (Cy3) or red (Cy5) signals, due to unbalanced ratio between the two fluorophores.

Probes located at +0.3 and -0.3 in the hg19 are in double copy (normal genotype); probes at +0.40 and +0.60 are in triple copy; more than +0.60 it is referred to gene amplification. Probes at -0.6 and 1 are in single copy, whereas probes at -2 are nullisomic.

### Immunohistochemistry

Surgical specimens are fixed in Carnoy, paraffin-embedded and sectioned at 2 μm. Sections are mounted on slides, paraffin removed by xylene and blocked in 10% H_2_O_2_ (Sigma-Aldrich, St. Louise, Missouri, USA). The sections are first incubated with normal goat serum (Dako, Glostrup, Denmark) and then in a humidified chamber with mouse monoclonal anti-EGFR (Thermo Scientific, INC, USA). Sections are then incubated with anti-mouse Envision® peroxidase conjugated (Dako, Glostrup, Denmark). Finally, sections reacted with diaminobenzidine (Liquid DAB Substrate Chromogen System, DakoCytomation Carpinteria, CA, USA), counterstained with hematoxylin and mounted.

For hematoxylin-eosin staining, slides deparaffined in xylene are stained in Carazzi hematoxylin solution, rinsed in running tap water and counterstain in eosin solution.

### Data analysis

The ΔΔCt method was used to determine gene expression and copy number variation; copy number variation (CNV) was determined as 2(2^- ΔΔCt^), where 2 is the usual copy number of a gene [24]. Numerical results were expressed as means ± SEM and statistical significance (P < 0.05) was evaluated using Student *t* test.

## Results

### Clinical and histological features

Tumor samples were obtained from 69 patients (48 males) operated at the IRCCS Foundation “C.Besta” Neurological Institute from 2007 to 2012. Mean age was 57 (range 18-78 years). 40/69 patients died after first surgery, 14/69 were re-operated after few months: the median survival time ranged from 9 to 12 months after surgery. Histologically, all tumors showed features of glioblastoma such as pleomorphic cells, microvascular proliferation, necrotic areas and pseudo-palisades.

### EGFR status in primary GBM and corresponding neurospheres

We performed a copy number variation (CNV) assay to assess the *EGFR* amplification status in 69 primary GBM (here defined as brain tumors, BT) and in corresponding neurospheres (here defined as NS), cultured in the presence of EGF and bFGF. To differentiate *EGFR* amplification from polisomy of chromosome 7 we considered the ratio between CNV of *EGFR* and *HGF*, both located on chromosome 7, normalised to the reference gene *TERT*.

Table 1A summarizes results in brain tumors (BT) with or without *EGFR* amplification and in corresponding NS: *EGFR* was amplified in 36 tumors (52%), but only in 23 corresponding NS (33%).

**Table 1:** **Summary of *EGFR *** **and *NFKBIA *** **copy number variation in primary GBM (BT) and corresponding neurospheres (NS)**

**1A**	***EGFR*** **wt**	***EGFR*** **amp**	**% amp**
**BT**	33	36	52%
**NS**	46	23	33%
**1B**	***NFKBIA*** **wt**	***NFKBIA+/-***	**% del**
**BT**	66	3	4%
**NS**	39	30	43%
**1C**	***EGFR*** **wt**	***EGFR*** **amp**	**loss of *EGFR *** **amplification**
***NFKBIA*** **+/- NS**	11	12	7

**Figure 1:**
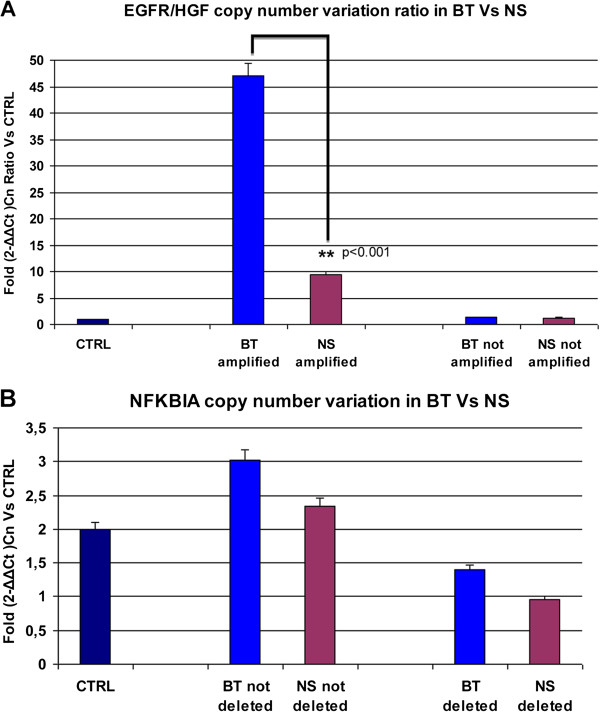
**Copy number variation values (CNVs) in primary GBM (brain tumors, BT) and corresponding neurospheres (NS). A)***EGFR* amplification in BT and corresponding NS is expressed as *EGFR/HGF* ratio in comparison to control DNA and shows significant loss of amplification in vitro (**p < 0.001). **B)** Heterozygous deletion in BT and NS shows a median copy number value of 1.2 compared to healty DNA and not deleted lines.

Figure 1A shows the *EGFR/HGF* ratio: in BT the median CNV ratio was 47; this value significantly decreased in NS, with median CNV ratio of 10 (p < 0.001).

Thus, 13/36 BT lost *EGFR* amplification in corresponding NS and *EGFR* CNV significantly decreased in the remaining 23 NS when compared to BT values.

### NFKBIA status in primary GBM and corresponding neurospheres

We then performed the CNV assay to assess the *NFKBIA* status on same BT and NS tested for *EGFR* amplification. Only 3 BT showed heterozygous deletion of *NFKBIA* and this alteration was mutually exclusive with *EGFR* amplification. Surprisingly, however, heterozygous deletion of *NFKBIA* was detected in a much larger number of cases, 30 of 69 NS lines (Table 1B).

The deletion was mutually exclusive with *EGFR* amplification in 18/30 NS; notably, 7/30 of these NS had lost *EGFR* amplification *in vitro*; in the remaining 12 NS the heterozygous deletion was present concurrently with *EGFR* amplification (Table 1C).

Figure 1B displays the median copy number of *NFKBIA*: combining BT and NS that we defined as hemizygously deleted for *NFKBIA* the median CNV was 1.2, while in remaining BT and NS the median CNV was 2.6.

To confirm that the CNV assay is able to distinguish one from two copies of a gene, we performed an assay to evaluate *HPRT1* CNV on 6 controls, 3 females and 3 males: *HPRT1* maps on chromosome X and therefore one copy only is present in males. Accordingly, females had 2 copies of the gene and males one copy only (Additional file 1: Figure S1), confirming that the CNV assay used to study *NFKBIA* detects hemizygosity.

**Figure 2:**
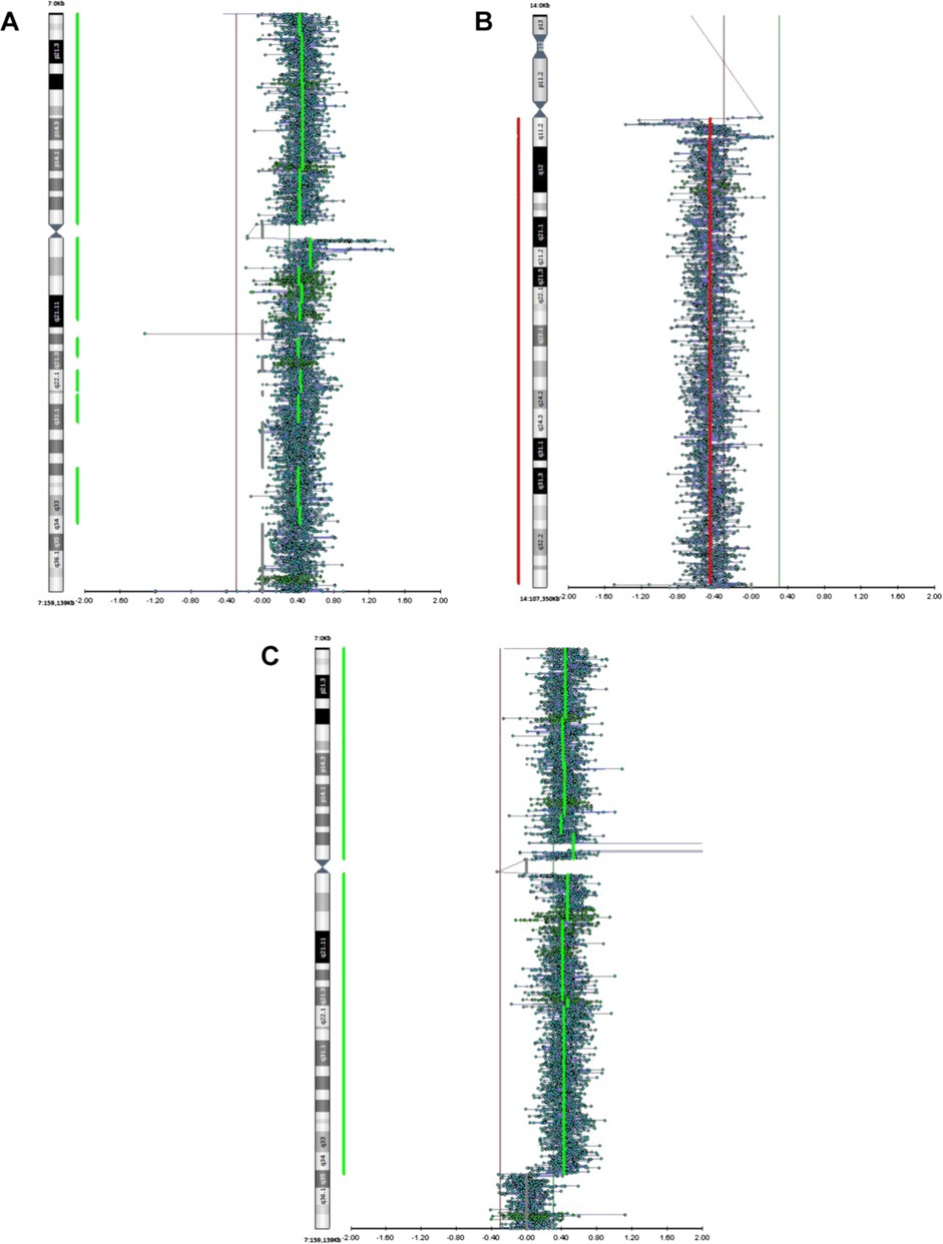
**Validation of CNV Assay by array-CGH analysis. A)** Trisomy of chromosome 7, but not *EGFR* amplification (probe position at +0.40), in BT314. **B)** Complete loss of chromosome 14q in BT314 (probe position at -0.40/-0.60); **C)***EGFR* amplification in primary tumor BT418 (probe position at +0.60).

Validation of CNV data also derived from array-CGH experiments on four GBM: BT418, 419, 314 and NS297. *EGFR* amplification on 7p11.2 (probe position according to hg19 at +0.60) was confirmed in two primary BT (BT418 e BT419) in which we found high *EGFR/HGF* ratios (74 and 323, respectively; BT418 shown in Figure 2C). BT314 showed trisomy of chromosome 7 and absence of EGFR amplification (probe position at +0.40) in agreement with results of the CNV assay (Figure 2A). BT314 showed *NFKBIA* deletion in the primary tumor: this was confirmed by array-CGH, showing complete loss of the long arm of chromosome 14 (Figure 2B). In NS297 *EGFR* amplification was pinpointed by the CNV assay but not by array-CGH, suggesting the possibility of a focal intragenic deletion not detectable by the CGH probes used in this array that only flank *NFKBIA*.

Taken together the data confirm that the CNV assay is able to detect gene amplifications and heterozygous deletions of *EGFR* and *NFKBIA*, respectively.

### Functional validation of NFKBIA heterozygous deletion

To validate further CNV data, we investigated the functional consequences of *NFKBIA* deletion. We speculated that the deletion could decrease intracellular levels of the NF-kB inhibitor (IkB), causing increased translocation of NF-kB into the nucleus and, consequently, increased transcription of target genes.

**Figure 3:**
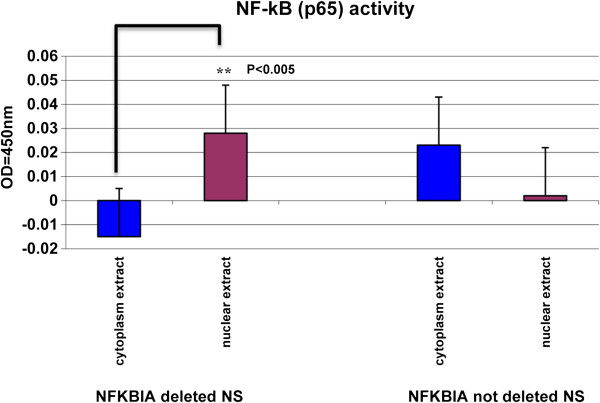
**Functional validation of *NFKBIA *heterozygous deletion in NS.** Comparison of p65 activity in cytoplasmatic versus nuclear proteins in NS shows a significant (** p < 0.005) activity only in nuclear extract of *NFKBIA* deleted NS.

As a readout of NF-kB activity we investigated p65 subunit activation. We compared p65 activity in cytoplasmic and nuclear extracts of 8 NS with *NFKBIA* deletion and 6 without deletion. As shown in Figure 3, we found that p65 activity was significantly higher in nuclear extracts of NS with *NFKBIA* deletion than in others (p < 0.005). This observation confirms that *NFKBIA* deletion in GBM NS may have relevant functional consequences.

**Figure 4:**
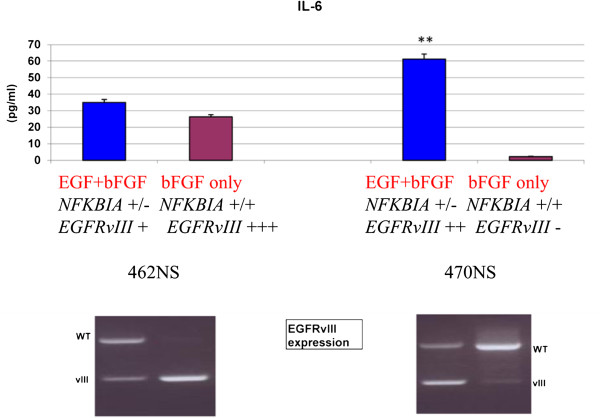
**IL-6 expression in cultured cells.** Quantification of IL-6 in surnatants of NS lines cultured in different conditions shows higher IL-6 production in BT470 in complete medium where N*FKBIA* deletion and EGFRvIII are both present (**p < 0.001). RT-PCRs displaying wild type *EGFR* and EGFRvIII are shown below for comparison.

As a further readout of NF-kB activity we also measured IL-6 levels in the culture medium of NS 462 and NS 470. This pro-inflammatory cytokine is a known target of NF-kB transactivation. Notably, IL-6 is also produced in EGFRvIII cells and released in the micro-environment to activate adjacent wtEGFR cells through GP130 activation [25]. IL-6 levels showed correlation with the presence of the EGFRvIII variant and *NFKBIA* deletion: they were present in NS 462 cells, cultured with or without EGF and in NS 470 cells cultured in complete medium, but were very low in the absence of EGF, when EGFRvIII is almost absent and NFKBIA is not deleted (Figure 4).

### Characterization of NS cultured with or without EGF

Previous data showed that EGF in the culture medium of NS may affect *EGFR* amplification [26]. To evaluate the contribution of EGF in the culture medium to *EGFR* amplification and /or *NFKBIA* expression, we established NS cultures from primary GBMs in the presence of either complete or modified medium (i.e. containing both EGF and bFGF or bFGF only, respectively).

**Figure 5:**
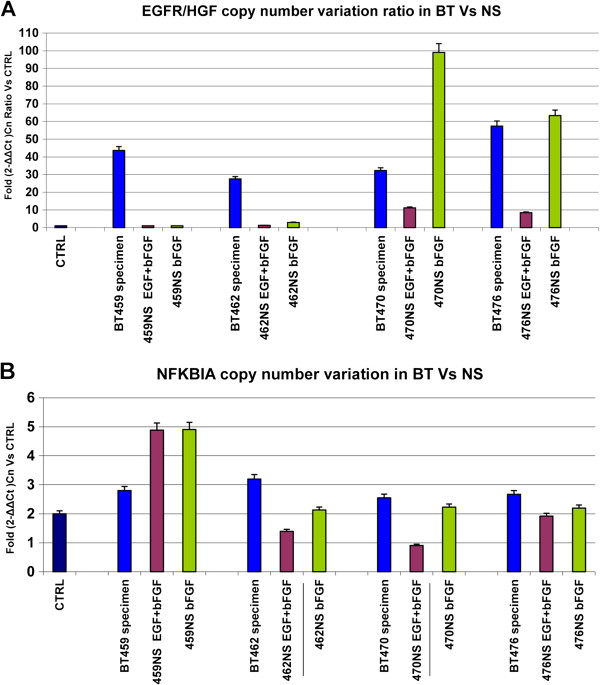
**Analysis of copy number variations (CNVs) in BT and corresponding NS cultured in different conditions. A)***EGFR* status in NS cultured in presence of both growth factors and in presence of bFGF only shows a strong CNV decrease in complete medium and persistence of *EGFR* amplification at variable levels in absence of EGF. **B)***NFKBIA* status in NS cultured in presence of both growth factors and in presence of bFGF alone shows an heterozygous NFKBIA deletion in two NS cultured in complete medium: BT462 and BT470.

We first investigated four NS lines deriving from BT with *EGFR* amplification. Figure 5 shows the comparison of *EGFR/HGF* copy number ratio (Figure 5A) and *NFKBIA* copy number (Figure 5B) in BT, NS in complete medium and NS in modified medium. In NS with complete medium all cell lines showed a marked decrease of *EGFR* amplification versus the corresponding BT, with 3 to 20 folds lower levels of *EGFR/HGF* copy number ratio. *EGFR* copy number was always higher in the absence of EGF: although this trend was present in all four cell lines, levels of *EGFR* amplification ranged from control values in NS 459 to levels higher than primary BT in NS 470 (Figure 5A).

Two cell lines, NS 462 and NS 470, showed heterozygous loss of *NFKBIA* in the complete medium, not detected in the original tumor: notably, the deletion was absent in the modified medium.

**Figure 6:**
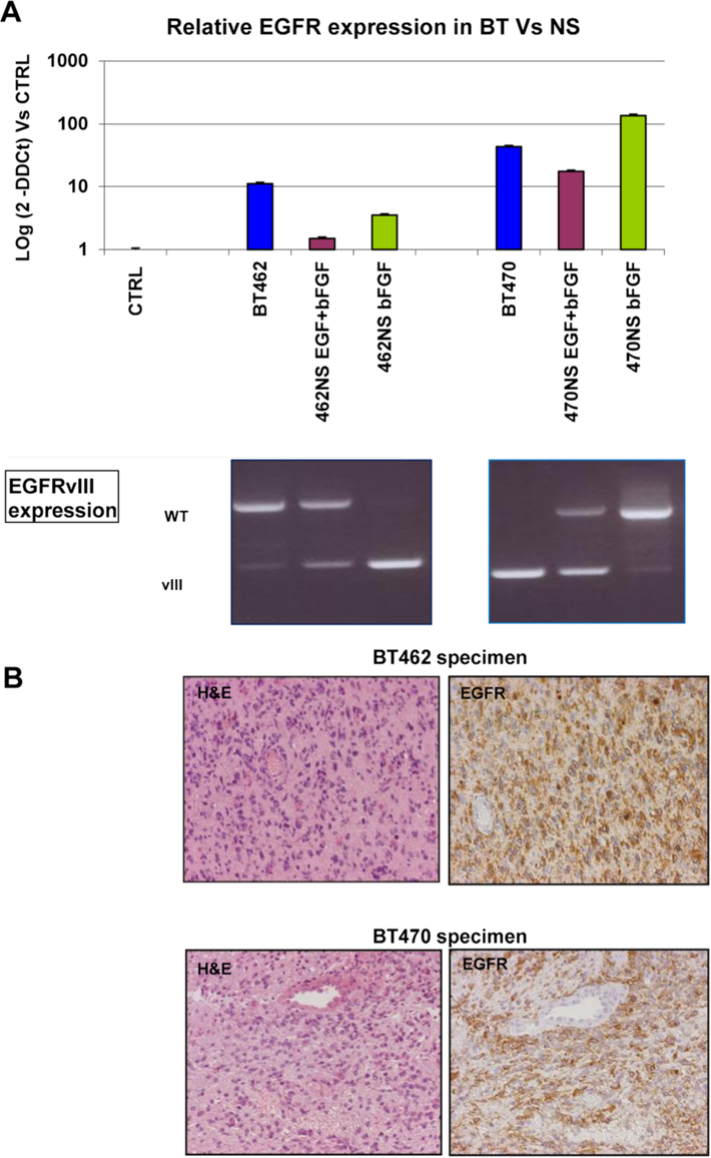
**EGFR and EGFRvIII expression in primary BT and corresponding NS cultured in different conditions. A)** The upper part shows mRNA levels of *EGFR* in primary tumors and corresponding NS; the lower part shows the presence of wild type *EGFR* and/or the mutant EGFRvIII: BT462 displays a gradual appearance of EGFRvIII in modified medium, whereas BT470 shows the presence of the mutant form in primary tumor and in NS cultured in complete medium; **B)** Immuno-histochemistry on tumors specimens shows high levels of EGFR in both tumors.

To further investigate the *EGFR* status we performed a quantitative evaluation of *EGFR* expression in BT and NS 462 and 470 by Real Time PCR, and investigated by a semi-quantitative method, RT-PCR, the presence of wild type *EGFR* and EGFRvIII (Figure 6A). The probe used for Real-Time PCR recognized both wtEGFR and EGFRvIII (see also Methods). NS 462 showed increased presence of EGFRvIII and decreased presence of wtEGFR, particularly in the absence of EGF. BT470, on the contrary, showed a strong decrease of EGFRvIII *in vitro* and an increase of wtEGFR, particularly in the absence of EGF.

In Figure 6B we show H&E staining and immunohistochemistry with anti-EGFR antibody of BT462 and BT470 indicating strong EGFR expression, as expected.

Two other cell lines were also considered, BT463 and BT467 that did not show *EGFR* amplification or *NFKBIA* loss in the original tumor. Under both culture conditions (EGF plus bFGF or bFGF only) the status of these two genes did not change, suggesting that absence of EGF *per se* is not sufficient to drive *EGFR* amplification (Additional file 2: Figure S2).

Overall the data suggest that loss of *EGFR* amplification *in vitro* is favored by EGF presence in the culture medium. Clonal expansion of cells harboring *NFKBIA* deletion and/or EGFRvIII deletion may take place in this context.

### Survival analysis

We looked for correlations of *EGFR* amplification and/or *NFKBIA* deletion and overall survival (OS): four GBM were excluded from analysis as the patients died early after diagnosis for reasons unrelated to tumor progression. We could not find significant correlations between the presence of *NFKBIA* deletion in NS and OS (Additional file 3: Figure S3A) also considering the association with presence or lack of *EGFR* amplification (Additional file 3: Figure S3B). *EGFR* amplification was also not associated with significant difference in OS (Additional file 3: Figure S3C), confirming ambiguities on the prognostic role of this marker [27].

## Discussion

### NFKBIA deletion is present at low frequency in primary GBM

In this work we investigated the presence of the *NFKBIA* deletion in GBM. After the report by Bredel et al [19] one paper only reported on *NFKBIA* deletions in a fraction of GBM but did not confirm mutual exclusivity with *EGFR* amplification [28]. Recent reports based on TCGA data did not mention a high frequency of hemizygosity on 14q13, where NFKBIA maps [29,30].

In our work we found a 4.3% frequency of NFKBIA deletion in 69 primary GBM. It is possible that the presence of normal cells in the surgical specimen leads to underestimation of the number of cases with deletion: this, however, seems plausible for a minority of cases. Furthermore, previous work reporting loss of heterozygosity on chromosome 14 showed loss of the entire q arm as a rather frequent event and partial losses located on 14q23-q32, telomeric to the NFKBIA locus [31,32].

It is plausible that other biological mechanisms independent of EGFR have a major role in NF-kB activation in GBMs. This family of transcription factors is the object of a number of studies pointing at its therapeutic targeting [33]. However its contribution to tumorigenesis in cells of neural origins needs further evaluation. Data from murine or rat NS showed that loss of both p65 and p50 NF-kB subunits results in a reduced number of progeny and in increased neuronal differentiation [34] and that activation of the canonical NF-kB pathway by TNF-alpha increases NS proliferation [35]. More recent data, however, suggest a pro-differentiation role of NF-kB. In murine embryonic stem cells Nanog, an essential factor for GBM tumorigenicity [36], binds to NF-kB proteins, inhibiting their transcriptional activity: overexpression of NF-kB proteins promoted differentiation, whereas inhibition increased expression of pluripotency markers [37]. Notably, also in GBM-NS the NF-kB transcriptional pathway was activated in the presence of serum-driven differentiation [38].

Overall, our first conclusion, based on this set of data is that the *NFKBIA* deletion is present but not frequent in primary GBM.

### NFKBIA deletion in culture is favored by EGF

This observation is strengthened by the *in vitro* data we gathered on GBM neurospheres (NS). Unexpectedly, we found a large fraction of GBM NS showing results of the copy number assay for *NFKBIA* compatible with a heterozygous deletion. In parallel we observed the disappearance of *EGFR* amplification in part of these NS and decreased CNV values in others. Our results suggest the possibility that *NFKBIA* deletions are magnified (or created) *in vitro* in GBM NS that have decreased or lost amplification of *EGFR*. Since EGFR signaling may also impinge of NF-kB, as indicated by data in breast cancer [16] and GBMs [18], it seems plausible that in tumors “addicted” to this signaling pathway, *NFKBIA* deletions arise or are selected for in the presence of loss or decreased amplification of *EGFR*. The idea that part of the *NFKBIA* deletions in NS are favored by culture conditions and therefore do not mirror the actual situation of the primary tumor receives some indirect support by the lack of correlations with OS that we have found (Additional file 3: Figure S3), differently from what reported by Bredel et al [19].

Two sets of observations suggest that the assay we used is appropriate for CNV studies in these cells. First, we validated this technique by showing that *HPRT1*, a gene located in chromosome X, is present with two copies in females and one copy in males (hemizygosity). Second, we showed that the decreased copy number of *NFKBIA* has functional consequences. We expected that decreased gene dosage of *NFKBIA* may cause increased migration to the nucleus of the NF-kB transcription factor and consequent increased activity of the p65 subunit. This was actually demonstrated in an ELISA-based testing on GBM NS with or without *NFKBIA* deletion. A third line of evidence was provided by array-CGH tested on one primary tumor, where *NFKBI*A deletion, detected by CNV assay, was associated to the entire loss of chromosome 14. *EGFR* amplification detected by CNV assay was also confirmed by array-CGH in these tumors and in one GBM NS. In this latter case, the *NFKBIA* deletion detected by the CNV assay was not found by array-CGH: we should consider, however, that the array CGH we used contain probes hybridizing to flanking and not intragenic regions of *NFKBIA*, so that hemizygosity would only be detected in the presence of large deletions or loss of the entire chromosome.

### EGFRvIII may be favored in vitro by lack of EGF

The progressive lack of *EGFR* amplification in the presence of EGF in the culture medium is in good agreement with data from Schulte et al, showing that *EGFR* amplification is progressively lost under culture conditions used for NS growth [26]. Notably these authors found that *EGFR* amplification is associated to slower growth *in vitro* and that the difference in growth is decreased/canceled if EGF is removed from the culture medium [26]. The recent observation that *EGFR* amplification is associated to increased capacity for invasion rather than proliferation is in agreement with this observation [39].

Another response that can be favored *in vitro* in the absence of EGF-induced signaling by EGFR is the partial deletion of *EGFR*, as this implies the loss of the extracellular domain interacting with its ligand and consequent constitutive activation [12]. EGFRvIII may also activate NF-kB through mTORC2 [7]: thus, this deletion, that occurs in the majority of GBMs with *EGFR* amplification [40], may also preserve the function of the EGFR-NF-kB axis in glioma perpetuation.

Previous data by different groups including ours, have proposed that GBM NS provide a better system than serum-dependent cultures, to study GBM biology *in vitro* and *in vivo*[22,23,41]. Present results, however, indicate that presence of EGF may partially skew the molecular signatures present in primary GBMs. This is well illustrated by recent data showing that TNF-mediated activation of NF-kB is stimulated by mcroglia-macrophages in the tumor microenvironment and lost in vitro, thus favoring a proneural phenotype in GBM NS [42].

In conclusion, the data we obtained indicate that *NFKBIA* deletions are present but not frequent in GBMs, at difference with the initial report by Bredel et al [19]. This information and available evidence from the literature suggest that more work is required to thoroughly define the relevant mechanisms of NF-kB activation in GBM.

## Competing interests

The authors declare that they have no competing interests.

## Authors’ contributions

MP performed CNV studies, investigated p65 activation and IL-6 expression and wrote preliminary results; PP, EB and SM cultured GB NS; GC performed the EGFR and EGFRvIII essays; VG, AR and FS performed CGH; ME provided clinical data; BP performed histological analysis; SP overviewed in vitro experiments; GF collabor ated to the study design and wrote the manuscript. All authors read and approved the manuscript.

## Supplementary Material

Additional file 1: Figure S1 Copy number validation assay. Validation of copy number variation assay to assess hemizygous or heterozygous condition versus wild type genotype. Click here for file

Additional file 2: Figure S2 Analysis of EGFR and NFKBIA copy number variations (CNVs) in primary tumors (BT) and corresponding neurospheres (NS) cultured in different conditions. Click here for file

Additional file 3: Figure S3 Kaplan Meier analysis of overall survival (OS) of 65 patients with GBM with relationship to NFKBIA deletion and/ or EGFR amplification. Click here for file
