# Supplementary figures and images for: Frequency of NFKBIA deletions is low in glioblastomas and skewed in glioblastoma neurospheres
Source: Mol Cancer. 2013 Dec 11;12:160. doi: 10.1186/1476-4598-12-160 (PMC4029392; doi:10.1186/1476-4598-12-160)

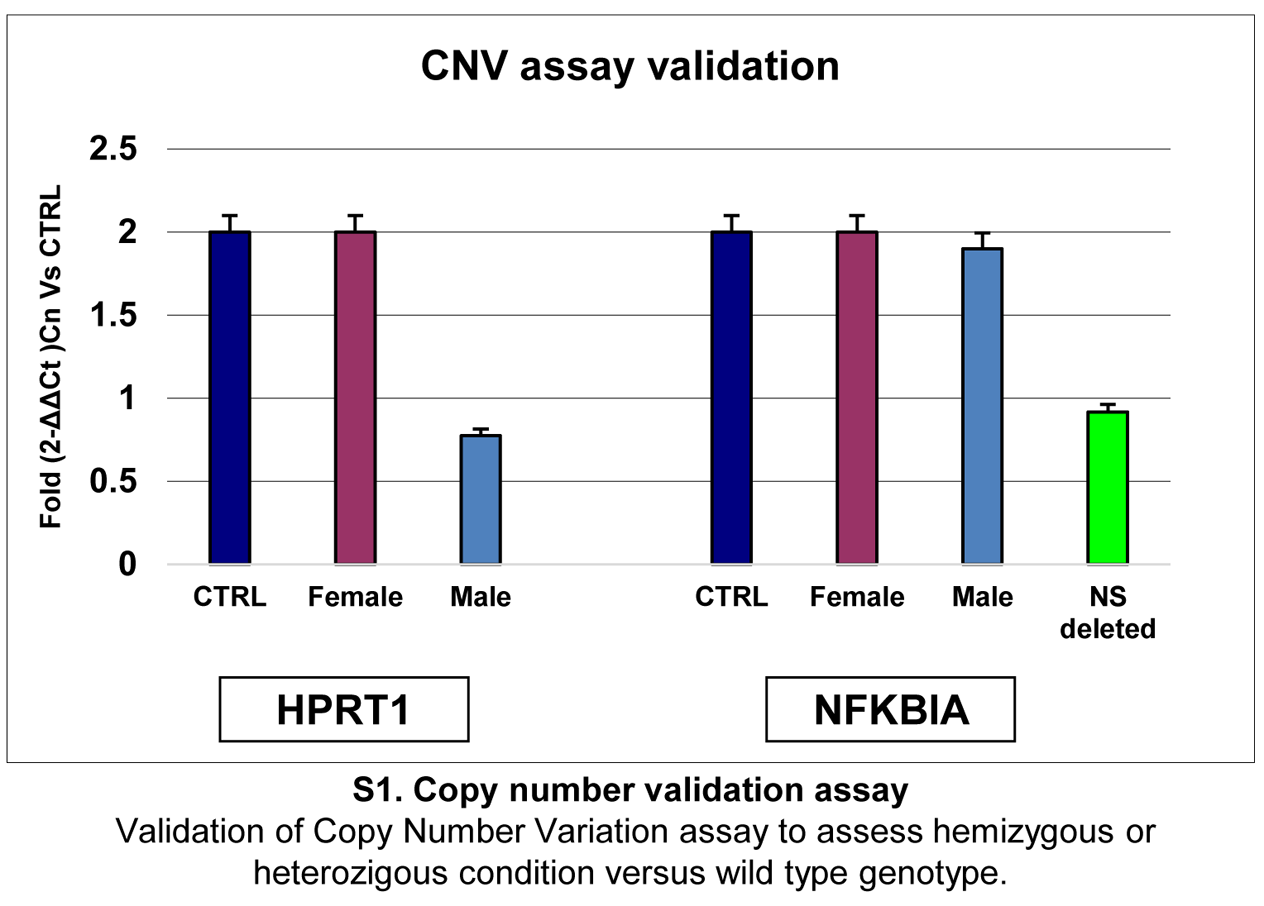

Supplement: Additional file 1: Figure S1 — Copy number validation assay. Validation of copy number variation assay to assess hemizygous or heterozygous condition versus wild type genotype. [file 1476-4598-12-160-S1.tiff]

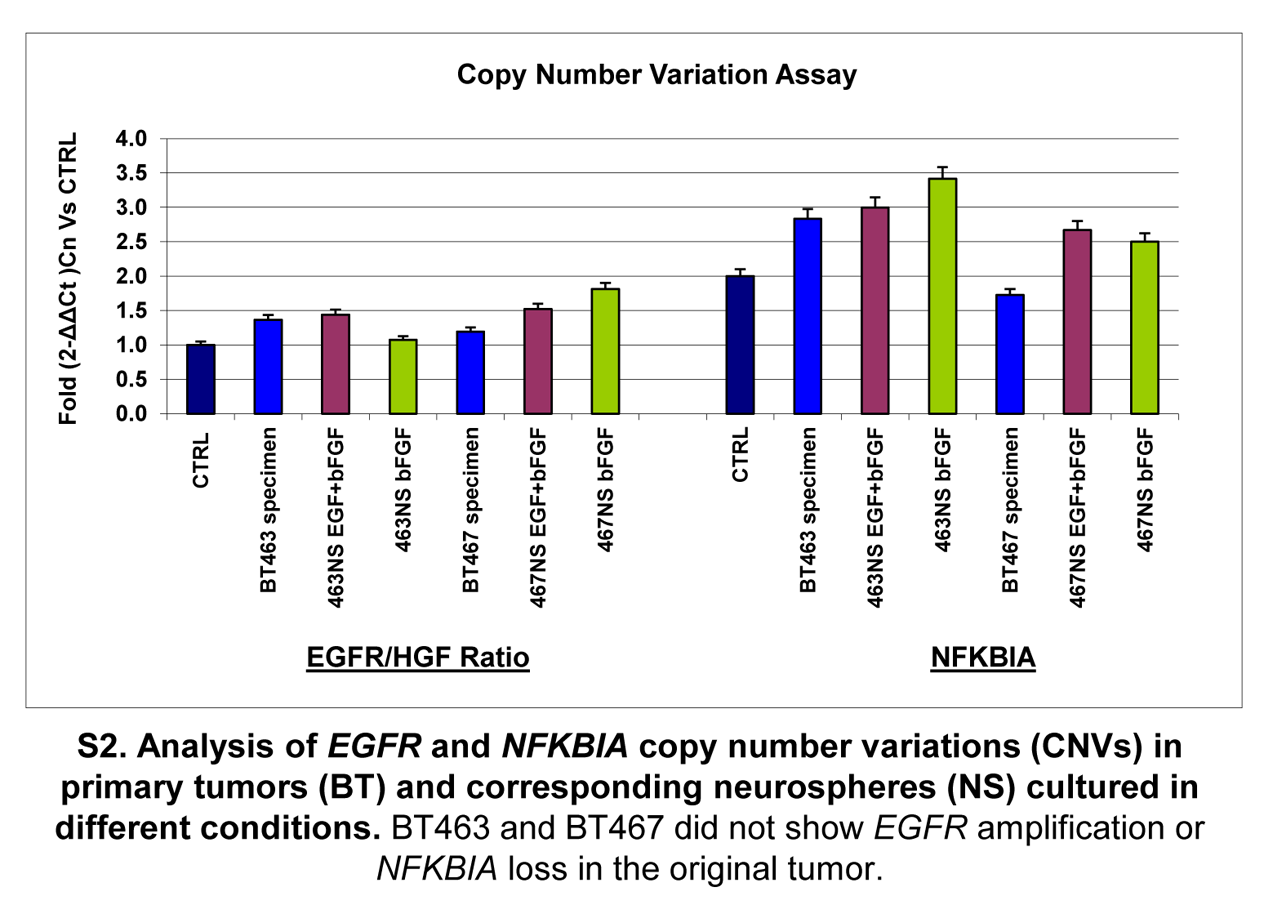

Supplement: Additional file 2: Figure S2 — Analysis of EGFR and NFKBIA copy number variations (CNVs) in primary tumors (BT) and corresponding neurospheres (NS) cultured in different conditions. [file 1476-4598-12-160-S2.tiff]

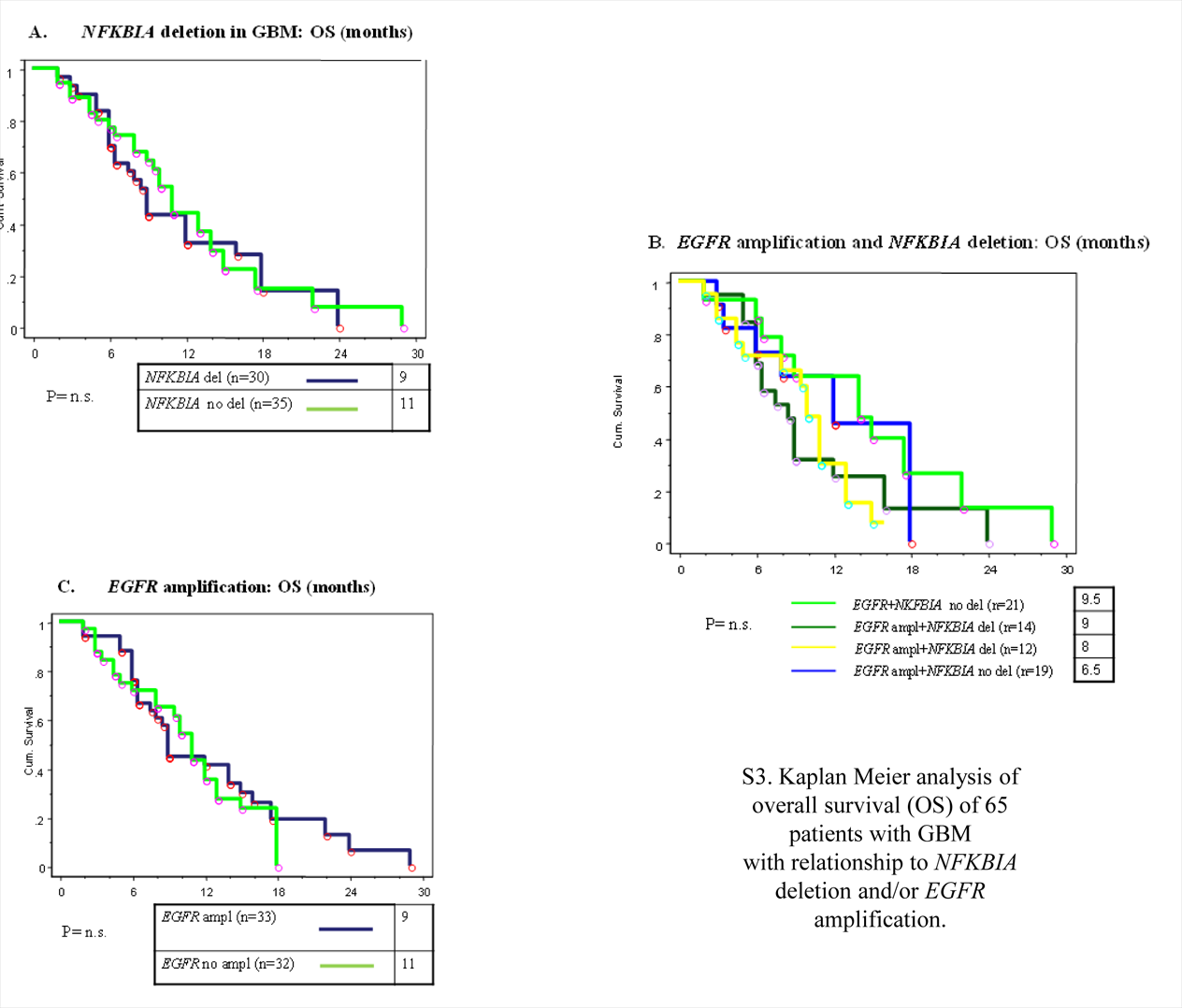

Supplement: Additional file 3: Figure S3 — Kaplan Meier analysis of overall survival (OS) of 65 patients with GBM with relationship to NFKBIA deletion and/ or EGFR amplification. [file 1476-4598-12-160-S3.tiff]
